# Some Notes on Local Anaesthetics

**Published:** 1910-04-15

**Authors:** John Harper


### SOME NOTES ON LOCAL ANAESTHETICS.

JOHN HARPER, M.R.C.S., L.R.C.P., L.D.S.

Amongst the many drugs which have been used to obtain local anaesthesia, cocaine will naturally present itself first, not alone on account of its potency but as being the earliest to come into general use in recent years; and had its action in actual practice been as localized as its advocates claimed, there would not have arisen the lengthy list of substitutes now obtainable. In dental work a sine qua non is that the patient shall exhibit no untoward symptoms after the use of any drug, and on this account the alarming and bizarre results in many cases of the injection of even small doses of cocaine have led to its abandonment by most of us.

Amongst the substances recommended as substitutes for cocaine may be mentioned stovaine, novocain, tropacocaine, betaeucaine, alypin, beta-eucaine lactate, nervanine and many others, and when estimating their local anaesthetic action Professor Braun's essentials should be taken into account, viz:—

1. A lower degree of toxicity than cocaine in proportion to its local anesthetic power.
2. Sufficient solubility in water. The solution should be stable and capable of sterilization by boiling.
3. Abscence of any sign of irritation. There should be no injury to the tissues, the local anaesthetic should be easily absorbed without causing any after effects, such as hyperemia, inflammation, infiltration or necroses.
4. Compatibility with adrenalin.
5. Rapid penetration of the mucous membrane and suitability for medullary anaesthesia.

All the drugs mentioned above and several others were recently investigated by Dr. Le Brocq, along the lines laid down by Professor Braun,. and I cannot do better than briefly mention the results obtained.:

Solubility in water:—Cocaine, stovaine, novocain, tropacocaine, beta-eucaine lactate, alypin, and nirvanine are fully soluble in water, their solutions are stable and as a 2 per cent. solution they will keep for a short time without deterioration.

Sterilization of solution:—Cocaine cannot be boiled, as decomposition occurs and the drug loses its activity; the others can be sterilized at 115 degrees C. if necessary and the drug is as active after as before sterilization.

Local anaesthetic action:—Cocaine being taken as the standard, stovaine has a more powerful anaesthetic action, weight for weight, than any of the rest.

Tozicity:—As the toxic action of these drugs is to paralyze the nervous system and so the respiratory centre, the toxicity in mammals must be regarded as the correct reading, for when respiration ceases the animal dies.

If the toxicity of cocaine be represented by 1, then that of

| | | | |
|---|---|---|---|
| Alypin | will | represent | 1.25 |
| Nirvanine | " | " | .714 |
| Stovaine (Chlorhydrete of Amylene) | " | " | .625 |
| Tropacocaine | " | " | .500 |
| Novocaine | " | " | .490 |
| Beta-eucaine | " | " | .414 |

Irritant Action on the Tissues:—When injected into the abdominal wall of rabbits, the skin being previously washed, shaved and made aseptic, cocaine was found to cause slight swelling and hyperaemia soon after injection.

Stovaine caused intense hyperaemia and dilitation of the blood vessels, followed by sloughing of the part.

Beta-eucaine lactate caused swelling and thickening about the seat of injection, followed by sloughing.

Tropacocaine caused swelling and some thickening, followed by sloughing.

Novocain showed no swelling and no hyperaemia, the part was perfectly normal after injection and remained so.

Compatibility with Adrenalin:—All the local anesthetics are compatible with adrenalin if the solutions are fresh and kept only for a short while. After a day or two the adrenalin decomposes unless it is kept in stoppered opaque bottles. The conclusion finally arrived at by Dr. Le Brocq was that of all the drugs investigated novocain is the most satisfactory for general use. Its anesthetic action is equal to that of cocaine and its toxicity and general destructive power on the tissues are very much less.

Speaking from personal observation after using stovaine, novocain, eudrenine, beta-eucaine, and cocaine I have had more satisfactory results from novocain than any of the others.

In regard to the technique of the injection of a local anaesthetic I need scarcely point out that asepsis as thorough as is possible when dealing with the oral cavity must be attended to. All syringes and needles, therefore, should be kept cleansed and the hands of the operator should be thoroughly washed before he proceeds to inject. The sterilizing glasses now obtainable, in which the syringe needle when not in use can be suspended in an antiseptic solution, are useful. A 5 per cent. solution of carbolic acid or lysol is an excellent medium, the latter being an efficient agent which has no action upon the metal of the syringe.

Many forms of syringe are now in use, the special requirements to which it should conform being, that it should be made entirely of metal, be simple in construction, powerful in action, and convenient to handle. There should be no glass about it to get fractured, and a transverse bar at the mouth of the barrel to serve as a fulcrum for the middle and index fingers of the operator, pressure being exerted by the thumb behind the piston. Screw-on needles are the best to use, and they should be of very fine calibre.

Before injecting, the mouth should be rinsed with an antiseptic mouth wash and the site selected swabbed over with a pledget of cotton wool dipped in an alcoholic solution of corrosive sublimate 1-1000 and then dried.

The point of insertion of the needle, most in favour, is midway between the gum margin and the position of the root of the tooth, in an oblique direction. A slow but gradual pressure on the piston of the syringe should lead to a blanching of the mucous membrane. When this blanching is well defined in outline, the needle after a short pause, should be slowly withdrawn and reinserted at the periphery of the blanched area, when more of the solution is again injected under pressure. By repetition of this procedure the whole of area surrounding the tooth or teeth to be extracted can be successfully rendered anaesthetic. In case of abcessed teeth care should be exercised not to inject into the pus cavity or marked sufferings will be caused. In order to avoid pain during the primary insertion of the needle a little ethyl chloride may be sprayed over the part where it is to enter, or some of the anaesthetic solution applied upon cotton wool for a short period before injecting.

Before concluding, I would like, if I may do so, to give you some account of a method not without interest, practiced by an English confrere, Dr. Parott, who makes use of local anaesthesia in consevative work. Dr. Parott's modus operandi is as follows: With a heavy metal syringe fitted with an ordinary sharp needle a preliminary injection is made into the gum, using perhaps not more than m.5 of the m.30 which the syringe contains. The needle is withdrawn and changed for a heavier one. A clean round bur, of a calibre corresponding to that of the heavy needle is placed in the handpiece and the head dipped in pure carbolic, any excess being shaken off. Tightening the soft tissues, if necessary, over the spot with a finger of the left hand, the bur, revolving fairly rapidly, is passed clean through them and made to penetrate the outer layer of compact bone, the perforation being made as near the level of the apices of the roots as easily practicable, in their direction and as nearly as possible midway between them, where the septum is thickest, to avoid injury of the periodental membranes. The yielding structure of cancellous bone is easily distinguished and the bur is withdrawn. If the preliminary injection has been effectively made, this small operation will be quite painless. The heavier needle is then inserted into the perforation in the bone under the soft tissues, the escharotic action of the pure carbolic on the bur, apart from its sterilizing action, aids materially in locating the perforation again, by whitening the edges of the puncture made by the bur in the soft tissues; without it there would be little visible sign of the perforation, owing to the automatic closing of the tissues and the comparative bloodlessness of the part from the preliminary injection.

The heavy needle is pressed well home into the part prepared for it and a deep injection made with the remaining contents of the syringe, care being taken that the fluid does not leak back; sometimes strong pressure is needed, and this must be guarded, as the fluid may find its way into any arterial or neural canal adjacent and entering with a rush may possibly cause a momentary pang to the patient, or a touch of nerves to the operator. Usually, however, it enters easily and steadily into the vascular bone.

In conclusion I trust, gentlemen, that these few notes, compiled without any pretentions to literary merit, may elict in your kindly criticism many points which will be of mutual benefit.—*Dominion Journal,* January 1910.

